# The complexities and caveats of lineage tracing in the mammary gland

**DOI:** 10.1186/s13058-016-0774-5

**Published:** 2016-11-25

**Authors:** Anne C. Rios, Nai Yang Fu, Joseph Cursons, Geoffrey J. Lindeman, Jane E. Visvader

**Affiliations:** 1 Stem Cells and Cancer Division, The Walter and Eliza Hall Institute of Medical Research, 1G Royal Parade, Parkville, VIC 3052 Australia; 2 Department of Medical Biology, The University of Melbourne, Parkville, VIC 3010 Australia; 3 Systems Biology Laboratory and ARC Centre of Excellence in Bio-Nano Science and Technology, Melbourne School of Engineering, University of Melbourne, Parkville, VIC 3010 Australia; 4 Bioinformatics Division, The Walter and Eliza Hall Institute of Medical Research, 1G Royal Parade, Parkville, VIC 3052 Australia; 5 Familial Cancer Centre, The Royal Melbourne Hospital and Peter MacCallum Cancer Centre, Grattan Street, Parkville, VIC 3050 Australia; 6 Department of Medicine, The University of Melbourne, Parkville, VIC 3010 Australia

## Abstract

Lineage tracing is increasingly being utilised to probe different cell types that exist within the mammary gland. Whilst this technique is powerful for tracking cells in vivo and dissecting the roles of different cellular subsets in development, homeostasis and oncogenesis, there are important caveats associated with lineage tracing strategies. Here we highlight key parameters of particular relevance for the mammary gland. These include tissue preparation for whole-mount imaging, whereby the inclusion of enzymatic digestion can drastically alter tissue architecture and cell morphology, and therefore should be avoided. Other factors include the scoring of clones in three dimensions versus two dimensions, the timing of induction, and the marked variability in labelling efficiency that is evident not only between different mouse models harbouring a similar gene promoter but also within a given strain and even within a single mammary gland. Thus, it becomes crucial to visualise extensive areas of ductal tissue and to consider the intricacies of the methodology for lineage tracing studies on normal mammary development and on potential ‘cells of origin’ of cancer.

A recent report by Wuidart and colleagues [1] describes several important limitations associated with lineage tracing strategies pertinent to the mammary gland. These include the nature of the transgenic or knock-in (KI) model, the degree of chimerism, and the need for a rigorous analysis of clones post-labelling. Additional considerations relevant to lineage tracing in the mammary gland are discussed below. Taken together with those raised in [1], these parameters likely account for disparities between lineage tracing data in the mammary gland biology field. Nonetheless, substantial evidence indicates the presence of both unipotent and bipotent mammary stem cells (MaSCs) (reviewed in [2]). It seems probable that bipotent cells help fuel the expansion of unipotent stem/progenitor cells when required and thus are biologically relevant.

## Preparation of whole-mounted mammary glands for three-dimensional (3D) confocal analysis

**Fig. 1:**
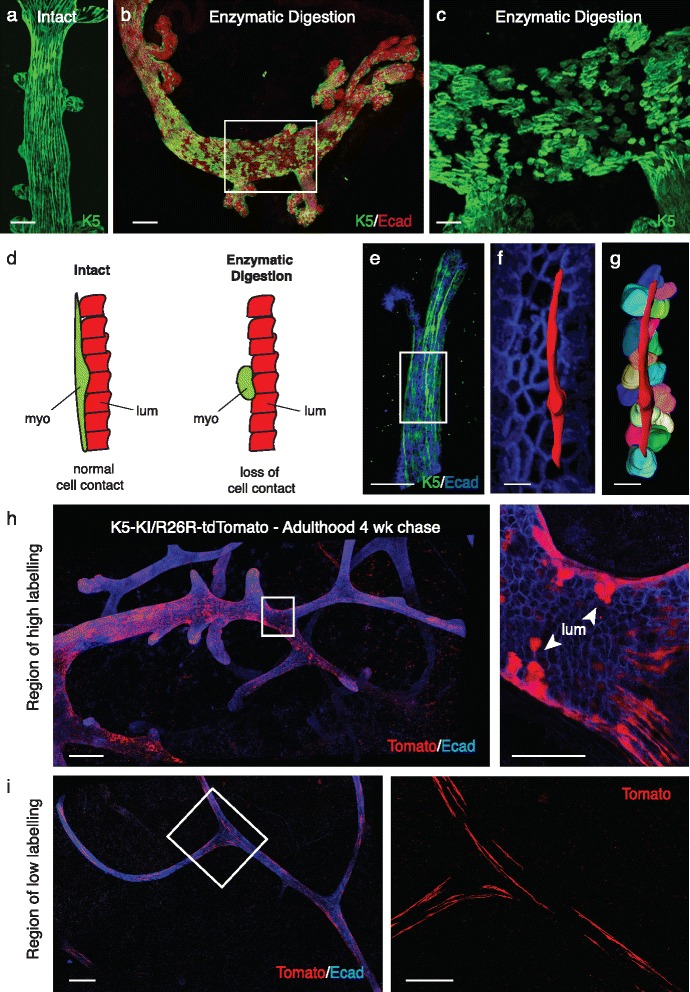
Influence of whole-mount preparation and efficiency of labelling on lineage tracing outcomes. **a** Whole-mount three-dimensional (3D) confocal image of an intact ductal portion immunostained for Keratin 5 obtained using a protocol that does not include any enzymatic digestion (described in Rios et al. [4]). *Scale bar* = 50 μm. **b** Whole-mount 3D confocal image of a ductal portion (8-week-old FVB/N mouse) immunostained for Keratin 5 (*green*) and E-cadherin (*red*), using the protocol described by Wuidart et al. for enzymatic digestion of mammary glands [1]. The mammary portions were subsequently fixed in 4% paraformaldehyde (PFA) for 2 h at 4 °C, and processed as described in Rios et al. [4] for immunostaining and confocal imaging. **c** Image of an enlargement from (**b**) showing the myoepithelial layer immunostained for Keratin 5. Note the paucity of myoepithelial cells and their altered shape, which is no longer elongated. *Scale bars* = 100 μm (**b**) and 30 μm (**c**). **d** Schematic panels showing normal contacts between one myoepithelial cell (*myo*, in *green*) and multiple luminal cells (*lum*, in *red*) in intact breast tissue (*left*), and loss of cell-cell contacts between the myoepithelial (in *green*) and luminal cells (in *red*) after enzymatic digestion (*right*). **e** Whole-mount 3D confocal image (from Fig. 1d, Rios et al. [4]) of a duct in a K5rtTA-IRES-GFP mammary gland immunostained for E-cadherin (in *blue*). **f** enlargement from (**e**), showing a mask (in *red*) applied to one myoepithelial cell (GFP channel using Imaris software) and the luminal layer labelled with E-cadherin (in *blue*). **g** Image showing the mask of the myoepithelial cell and the masks of all luminal cells in direct contact with the myoepithelial cell. Note: the mask was applied to a representative 70 μm myoepithelial cell. *Scale bars* = 50 μm (whole-mount **e**) and 10 μm (enlargements **f**, **g**). **h, i** Whole-mount 3D confocal images of ducts in a K5-KI/R26R-tdTomato mammary gland, showing either a high (**h**) or low (**i**) degree of labelling in the same gland, at 4 weeks post-induction with tamoxifen in adulthood and then immunostained for E-cadherin (*blue*). Enlargements show optical sections depicting luminal cell-containing clones (*upper panel*) or elongated myoepithelial cells (*lower panel*). *Scale bars* = 200 μm (whole-mounts) and 50 μm (enlargements)

Protocols that utilise proteolytic digestion [1] may affect tissue organisation and compromise mammary ductal tree integrity. Comparison of tissues prepared with and without enzymatic digestion revealed that even low concentrations of proteolytic enzymes can lead to a striking depletion of cells in the outermost myoepithelial layer (Fig. 1a–c), evident as gaps. Thus, destruction of the basal lamina by enzymatic digestion appears to profoundly change the morphology of myoepithelial cells such that they lose their characteristic elongated shape and become rounded (Fig. 1b and c). As a consequence, their physical interactions with luminal cells can be lost, with significant implications for clonality studies (Fig. 1d). Tissues that lack elongated cells such as the prostate [3] may not show sensitivity to the same digestion conditions. Indeed, Wuidart et al. [1] could readily track bipotent stem cells in the prostate under the same preparation conditions.

## Clonal analysis in 3D

It is advantageous to score clones in 3D to establish whether cells are directly adjacent [4]. A luminal and myoepithelial cell may be touching in 3D but this is not always apparent within 2D data. Myoepithelial cells span 100 microns while luminal cells are smaller and a single myoepithelial cell can contact multiple luminal cells. Remarkably, a single 70 micron myoepithelial cell can even contact 17 luminal cells (Fig. 1e–g). Visualisation of the four colours of the Confetti locus by native fluorescence in 3D allows scoring of around 100 clones in 1–2 days in a rather laborious procedure. In the clonal analysis of K5rtTA/TetO-cre/R26R-Confetti mice at 1 week post-induction in the adult [4], the identification of unicoloured clusters comprising both myoepithelial and luminal cell types was clear, while abutting luminal and myoepithelial cells of different colours were very rare in tilescans that covered millimetres of contiguous ducts, underscoring the importance of 3D imaging to identify bipotent stem cells.

Using 3D confocal imaging, similar data were obtained and enumerated for the K14creER model addressed in Wuidart et al. [1]. In K14creER/R26R-YFP glands, we observed 1–2% labelled luminal cells versus ~40% basal cells at 2 days post-induction in the adult [4] (unpublished data). No labelling of luminal cells occurred prior to induction. Analysis of sparsely populated ducts in K14creER/R26R-Confetti mice pulsed in adulthood for 8-weeks revealed 49% myoepithelial-only, 48% mixed, and 3% luminal-rich mixed clones. To directly address the probability of unicoloured vs multicoloured doublets/clusters, we enumerated *all* cells and clusters (2–5 cells) in a ductal region of K14creER/R26R-Confetti mammary glands that displayed very sparse labelling (unpublished data). Scoring revealed 93.4% unicoloured (including bilineage) vs 6.6% multicoloured clusters. The majority of cells scored in 3D corresponded to single myoepithelial cells, and the next most represented group was myoepithelial doublets/triplets of the same colour. In the case of multicoloured clusters, it should be noted that there was one comprising a GFP^+^ (nuclear) luminal cell and a different coloured myoepithelial cell, but that a GFP^+^ myoepithelial cell was located in very close proximity (one cell away). If the null hypothesis is that clusters are equally likely to be the same or different colours, then the two-sided *p* value is 8.7e^–7^ (using an exact binomial test). Thus, the presence of unicoloured bipotent clones is statistically significant. Importantly, no luminal-only clones were detected in the K5rtTA, K14creER, or huK5creER/YFP models [4]. Collectively, these 3D data argue against chance (or ‘leaky’) induction of neighbouring unipotent luminal progenitors in these basal models.

## Efficiency of cell labelling

The efficiency of labelling is crucial, as indicated by Wuidart et al. [1], and different strategies have been applied between groups. We aimed for a more substantial degree of labelling to allow detection of stem cells since they are estimated to comprise about 5% of the heterogeneous basal population [5, 6], and Keratin (K)5 and K14 mark the entire basal population. The level of labelling varied dramatically between models, between individual mice of a given strain but also within a single mammary gland. Even when the labelling frequency was 30%, not all areas of the tree exhibited the same degree of labelling. A striking example of this variation is shown in Fig. 1h and i, where two regions from the same mammary gland of a K5-KI/R26R-tdTomato mouse show differences in labelling. Although predominantly expressed in the basal lineage as originally reported [7], clear evidence for bipotent stem cells was obtained upon a higher degree of labelling (using a low dose of tamoxifen). In branches comprising fewer labelled cells, only K5^+^ myoepithelial cells were evident, perhaps owing to insufficient *cre* expression to activate recombination. FACS analysis of K5-KI/R26R-tdTomato mice (>6) confirmed that 10–15% of Tomato^+^ cells corresponded to luminal cells when the labelling efficiency of the basal population was greater than 60%. Given the observed variation in labelling between different ducts within the same mammary gland, and likely differences amongst stem cells in different regions, it is essential to visualise millimetres of ductal tissue in 3D. In addition, tamoxifen continues to be a concern as the field uses varying doses of tamoxifen, which can affect labelling efficiency and read-out, given its pronounced effect on the basal and luminal lineages [4, 8].

A labelling efficiency of around 100% has been recently reported for the K14rtTA model [1], with labelled progeny restricted to unipotent myoepithelial stem cells. We agree that this model is largely unipotent. However, 3D imaging of millimetres of ductal tissue from K14-rtTA/TetO-cre/R26R-tdTomato mice (achieving 89% saturation labelling of basal cells) revealed the presence of rare bi-lineage clones comprising directly abutting luminal and myoepithelial cells, thus arguing against nonspecific labelling (data not shown).

## Timing of induction for clonal analyses

Mammary gland development is relatively complex and occurs in multiple phases. Therefore, an additional consideration for mammary gland lineage tracing strategies is the timing of labelling induction for clonal analyses. MaSC populations will inevitably differ in their behaviour and location during development (embryonic and pubertal) versus the adult stage (ductal maintenance and remodelling during reproduction) due to different physiological requirements. For clonal analysis, induction should be performed during the adult stage and not in puberty. This is due to the nature of elongation that occurs from the highly proliferative, multilayered terminal endbuds during puberty and the movement of cells to form the subtending duct that preclude the spatial identification of clones during this morphogenic stage.

## Implications for data quantification

Wuidart et al. [1] recently established a statistical model that describes the relative frequency of adjacent unicolour pairs (UPs) within tissue data. However, we believe that proteolytic digestion applied for tissue processing [1] has the potential to introduce artefacts into the quantitative data used to inform their statistical model, since it affects the morphology of myoepithelial cells and thus the number of neighbouring cells. The myoepithelial cell shown (Fig. 1e–g) contacts 17 luminal ductal cells, greatly exceeding the maximum value of 7 reported by Wuidart et al. Although an increase in the number of neighbouring cells will increase the number of adjacent UPs expected by chance, it would also greatly increase the observed number of UPs, highlighting the complex effects of tissue preparation upon data quantification. In addition, the model does not account for the low frequency of labelling of MaSCs compared to other cell types in the mouse models examined. MaSCs are also unlikely to be homogeneously distributed throughout the ductal tree (unpublished data). In the future, mathematical modelling will provide a powerful means to interrogate cellular dynamics and the spatial localisation, but care must be taken when interpreting the image data and quantifying model parameters. More extensive image data obtained using refined genetically engineered mouse strains that allow different populations to be marked will help guide the development of such models.

## Concluding remarks

It is clear that the precise *cre* strain, even using similar transgenic promoter segments, can drastically influence the outcome of lineage tracing studies. Moreover, a negative result in lineage tracing does not prove that a cell type does not exist. Rather, it can reflect the *cre* driver or methodology employed. To track MaSCs in vivo, the field requires highly specific gene promoters for these cells. The *Lgr5* gene has been previously shown to be basally restricted in the adult [9–11] and to mark a subset of basal cells that includes bipotent MaSCs [4]. Wuidart et al. [1] now report that up to 40% of labelled cells correspond to luminal cells yet conclude that *Lgr5* does not mark any bipotent cells. *Procr* is also more restricted than the basal keratin genes and has been shown to mark bipotent stem cells by lineage tracing [12]. Interestingly, it has proven difficult to track hematopoietic stem cells in vivo using lineage tracing methods, but it is widely accepted that these cells are critical for blood development and cancer [13, 14]. We believe that the overarching question for the mammary gland field is not whether bipotent stem cells exist, but instead what is the relative contribution of bipotent versus unipotent stem cells to the different stages of post-natal mammopoiesis?

## Abbreviations

K: Keratin
KI: Knock-in
Lgr5: Leucine-rich repeat-containing G-protein-coupled receptor 5
MaSC: Mammary stem cell
Procr: Protein C receptor
UP: Unicolour pair
